# Durable response of primary cardiac lymphoma after autologous stem cell transplantation and sequential CAR-T therapy: a case report and literature review

**DOI:** 10.3389/fimmu.2025.1581654

**Published:** 2025-08-28

**Authors:** Ge Wang, Xiaoxi Zhou, Pengcheng Wang, Zekai Mao

**Affiliations:** ^1^ Department of Gastroenterology, Tongji Hospital, Tongji Medical College, Huazhong University of Science and Technology, Wuhan, China; ^2^ Department of Hematology, Tongji Hospital, Tongji Medical College, Huazhong University of Science and Technology, Wuhan, China; ^3^ Immunotherapy Research Center for Hematologic Diseases of Hubei Province, Tongji Hospital, Tongji Medical College, Huazhong University of Science and Technology, Wuhan, Hubei, China; ^4^ Emergency Center, Hubei Clinical Research Center for Emergency and Resuscitation, Zhongnan Hospital of Wuhan University, Wuhan, China

**Keywords:** primary cardiac lymphoma, CAR-T therapy, autologous hematopoietic stem cell transplantation, pericardial effusion, chemotherapy

## Abstract

Primary cardiac lymphoma (PCL), an exceedingly rare and aggressive extranodal lymphoma confined to the heart and/or pericardium, poses significant diagnostic and therapeutic challenges with a historically dismal prognosis. We present the first documented case of PCL achieving sustained complete response (CR) through a novel therapeutic approach combining autologous hematopoietic stem cell transplantation (ASCT) with sequential tandem CD19/20 CAR-T therapy. A 55-year-old female presented with chest tightness and pericardial effusion in April 2023. The diagnostic process involved multiple modalities and analyses, culminating in a diagnosis via CT-guided mediastinal lymph node biopsy. The patient underwent several chemotherapy regimens, including combinations of rituximab and polatuzumab vedotin, R-CHOP, Pola-R-CHP, and Pola-R-DA-EPOCH, which led to symptom relief and partial response. Subsequently, ASCT was performed, followed by sequential tandem CD19/20 CAR-T therapy. As a maintenance treatment, the PD-1 inhibitor sintilimab was administered approximately every two months. The patient achieved CR at month 3 and maintained CR for one year following the CAR-T infusion. Following CAR-T therapy, the patient developed immune effector cell-associated hematologic toxicity, which was managed with granulocyte colony-stimulating factor injection and supportive care. This case illustrates the difficulties in diagnosing and treating PCL. Through the combination of ASCT and sequential tandem CD19/20 CAR-T therapy, the patient achieved sustained CR with manageable toxicity. This case demonstrates the feasibility, safety, and potential efficacy of this combination strategy in treating high-risk lymphoma. Moreover, we propose a structured algorithm that may help optimize the clinical implementation of CAR-T therapy in similar cases. Continued follow-up and broader studies are warranted to validate these findings.

## Introduction

Primary cardiac tumors are extremely rare, with an incidence of approximately 0.001%–0.03% in the general population. The majority are benign, whereas approximately 25% are malignant, including sarcomas, mesotheliomas, and primary cardiac lymphomas (1). Cardiac lymphoma represents a rare subset of extranodal lymphomas, typically diagnosed incidentally or during evaluation for nonspecific cardiac symptoms. Compared with secondary cardiac lymphoma (SCL), primary cardiac lymphoma (PCL) is exceedingly rare, contributing to the diagnostic complexity in diagnosis and urgency in treatment (2). Here, we present the first documented case of PCL achieving sustained complete response (CR) through a novel therapeutic approach combining autologous hematopoietic stem cell transplantation (ASCT) with sequential tandem CD19/20 chimeric antigen receptor T-cell (CAR-T) therapy.

## Case presentation

A 55-year-old woman presented to Tongji Hospital, Tongji Medical College, Huazhong University of Science and Technology, in April 2023 with intermittent chest tightness persisting for one month. She had no significant past medical history and denied smoking, alcohol use, or a family history of malignancy or cardiovascular disease. At symptom onset, she experienced nocturnal chest tightness, worsened by back pain, referred pain in both shoulders, and episodes of nausea and vomiting, especially during severe attacks. She denied fever, cough, headache, dizziness, abdominal pain, or bloating. Physical examination revealed a weight of 55 kg, a height of 158 cm, and a heart rate of 120 bpm. No other abnormal findings were observed. Laboratory tests revealed elevated levels of high-sensitivity troponin (0.03 ng/mL), N-terminal pro-B-type natriuretic peptide (NT-proBNP, 723 pg/mL), erythrocyte sedimentation rate (40 mm/h), and C-reactive protein (10.78 mg/L), with no obvious abnormalities in routine blood tests, thyroid function, or tumor markers. A T-spot test was performed to exclude infection with *Mycobacterium tuberculosis*, and the result was negative. Electrocardiogram (ECG) showed sinus tachycardia, and coronary computed tomography (CT) angiography showed no stenosis. Chest CT showed pericardial effusion and enlarged mediastinal lymph nodes. Echocardiography demonstrated significant pericardial effusion involving various cardiac walls, with thicknesses measuring 21 mm in the left ventricular posterior wall, 9 mm in the right ventricular anterior wall, 7 mm in the apex, 20 mm in the left ventricular lateral wall, and 21 mm in the right atrial wall. Assessment of cardiac function revealed a left ventricular ejection fraction (EF) of 65% and increased left ventricular end-diastolic pressure.

Diagnostic pericardiocentesis and subsequent drainage of pericardial effusion were performed at a local hospital in Henan Province on 6 and 13 April, respectively. Given normal levels of adenosine deaminase and tumor markers in the pericardial fluid, along with the absence of notable abnormalities in fluid biochemistry and exfoliative cytology, the exact cause of the pericardial effusion remained elusive. Initially suspected to be tuberculosis, the patient was started on anti-tuberculosis treatment. Follow-up echocardiography on 22 April showed a marked reduction in the thickness of the pericardial effusion.

**Figure 1:**
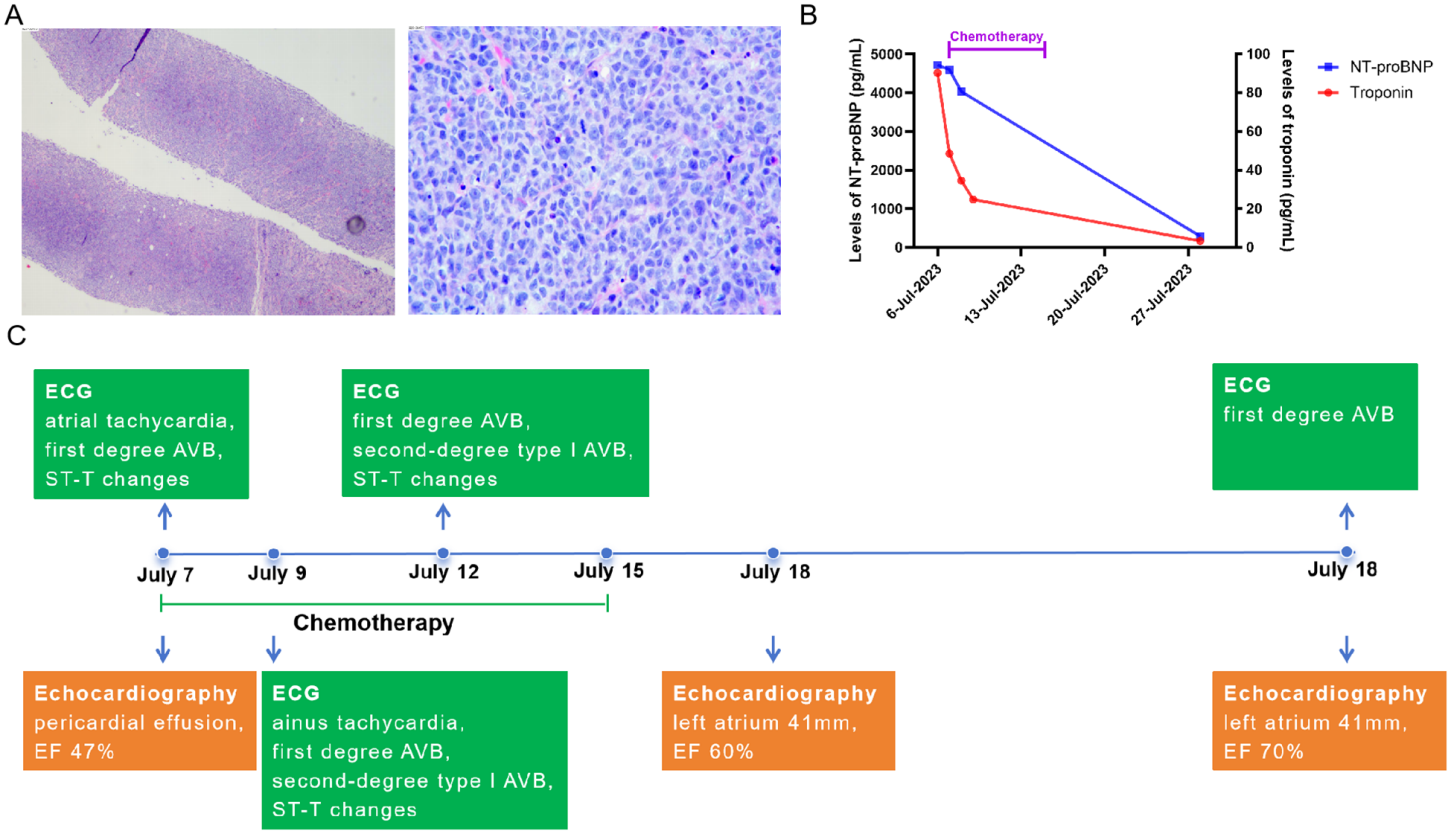
The pathology of primary lymphoma and changes in cardiac indicators after the first chemotherapy. **(A)** the pathology of primary lymphoma, the biopsy was from mediastinal lymph node puncture. **(B)** changes of levels of NT-proBNP and troponin before and after first chemotherapy in this case. **(C)** changes of ECG and echocardiography after the first chemotherapy. AVB, atrioventricular block; EF, Ejection Fractions; TB, tuberculosis; DLBCL, diffuse large B-cell lymphoma.

However, one week later, the pericardial effusion increased, and the patient again experienced chest tightness. Cardiovascular magnetic resonance (CMR) revealed pericardial thickening with heterogeneous enhancement, diffuse abnormal signals in the left and right ventricular myocardium, pericardial effusion, and a small left-sided pleural effusion. A positron emission tomography-computed tomography (PET-CT) on 3 May identified metabolically active soft tissue densities in the pericardial and mediastinal areas (SUVmax 18.9), suggesting malignancy. Multiple enlarged lymph nodes with high metabolic activity were evident, particularly in mediastinal zones 2 through 6 (size 1.8 cm ∗ 2.2 cm, SUVmax 16.2). However, subsequent fiberoptic bronchoscopy, alveolar lavage, and transbronchial lung biopsy yielded negative results. Consecutive examinations of exfoliative cytology from pericardiocentesis and drainage of pericardial effusion also showed no evidence of malignancy. With administration of anti-tuberculosis treatment and diuretics, the patient’s chest tightness initially improved but soon worsened due to increasing pleural effusion, measuring 71 mm on the left side and 64 mm on the right. The patient was referred to a hospital in Beijing for further diagnosis and treatment in June. Diagnostic thoracentesis and drainage of the pleural effusion were performed, indicating transudate with negative culture results and negative GeneXpert MTB/RIF assay result, an automated molecular test for *Mycobacterium tuberculosis* (MTB) and rifampin (RIF) resistance (3). Flow cytometry results identified suspicious abnormal clonal B-cells, characterized by positive expression of CD45, CD19, CD20, CD5, CD38, CD22, Bcl-2, and Ki-67 (positive in 78.5% of cells), and negative expression of CD2, CD3, CD4, CD7, CD8, CD30, kappa, lambda, cytoplasmic IgM, and CD79b. Contrast-enhanced chest CT performed on 12 June showed multiple soft tissue densities in the mediastinum, bilateral hilar zones, peri-aortic and pulmonary artery regions, pericardium, and increased pleural and pericardial effusions, suggesting disease progression and malignancy. A CT-guided mediastinal lymph node biopsy was performed following a multidisciplinary team (MDT) discussion. The biopsy findings, along with immunohistochemical analysis, revealed a predominance of B-cell markers (CK (–), Vimentin(−), LCA(+), CD5(+), CD117(−), TdT(−), TTF-1(−), P40(−), CD20(+), CgA(−), Syn(−), CD56(−), CD1a(−), CD3(−), PAX-5(+), MuM1(+), Bcl6(+), CD10(−), C-MYC(20%+), BCL2(SP66)(+), Ki-67[positive in 90% of cells]), confirming the diagnosis of non-Hodgkin’s lymphoma (**Figure 1A**). The result was consistent with diffuse large B-cell lymphoma (DLBCL), non-germinal center B-cell-like (non-GCB) subtype, according to the 2015 World Health Organization (WHO) Classification of Cardiac and Pericardial Tumors (4). Upon diagnosis, the patient was promptly transferred back to Tongji Hospital for continued management on 6 July 2023. The pathological diagnosis was independently confirmed by the Department of Pathology, Tongji Hospital, using both hematoxylin–eosin staining and comprehensive immunohistochemistry. Fluorescence *in situ* hybridization analysis revealed no rearrangements of the BCL6, BCL2, or c-MYC genes, and no deletion of the TP53 gene was observed. The patient denied any neurological symptoms, and head magnetic resonance imaging showed no abnormalities.

Given the patient’s severe cardiac symptoms at presentation and potential intolerance to standard-intensity chemotherapy, she was started on a first-line regimen combining cyclophosphamide (200 mg iv drip d1, 400 mg iv drip d2–4), rituximab (600 mg iv drip d4), polatuzumab vedotin (Pola, 90 mg iv drip d6), and dexamethasone (15 mg iv drip d1–5) on 7 July. This stepwise therapy led to temporary symptom relief and a reduction in pericardial effusion. Additionally, the patient’s myocardial injury biomarkers, cardiac function, and arrhythmias improved rapidly, with EF increasing from 47% to 60% (**Figures 1B, C**). The patient received two cycles of R-CHOP (rituximab 600 mg d1, cyclophosphamide 1,000 mg d2, liposomal adriamycin 40 mg d3, vincristine 4 mg d4, dexamethasone 15 mg d1–6) started from 29 July to 25 August, followed by Pola-R-CHP (rituximab 600 mg iv drip d1, Pola 90 mg iv drip d2, cyclophosphamide 1,000 mg iv drip d3, doxorubicin 50 mg iv drip d3, dexamethasone 15 mg d3–7) therapy from 25 September. Subsequently, she received intensive immunochemotherapy—Pola-R-DA-EPOCH chemotherapy (rituximab 600 mg iv drip d1, Pola 90 mg iv drip d2, etoposide 70 mg iv drip d3–6, doxorubicin 20 mg iv drip d3–6, dexamethasone 15 mg bid d3–7, cyclophosphamide 1,000 mg iv drip d7)—from 17 October to prepare for stem cell mobilization. Hematopoietic stem cells were collected successfully. Imaging evaluation showed that the patient achieved a partial response after chemotherapy. These chemotherapy regimens were well tolerated. However, chest CT performed on 15 September revealed a pulmonary infection, and pathogen screening identified *Pneumocystis jirovecii*. After treatment with trimethoprim-sulfamethoxazole and micafungin, *P. jirovecii* nucleic acid turned negative, and subsequent chest CT showed improvement. Following Pola-R-CHP treatment, the patient developed bone marrow suppression and high fever, which improved after granulocyte colony-stimulating factor (G-CSF) therapy and anti-infective treatment. On 21 November, she received another cycle of rituximab combined with polatuzumab vedotin as bridging treatment.

To continue treatment for PCL, the patient was enrolled in an investigator-initiated trial (IIT) study as second-line therapy conducted by Tongji Hospital, Tongji Medical College, Huazhong University of Science and Technology. The study was approved by the Institutional Review Board of Tongji Hospital and registered with the Chinese Clinical Trial Registry (registration number NCT05618041). Informed consent was obtained after detailed discussion with the patient regarding risks and expected outcomes. Briefly, following T-cell apheresis on 20 November 2023, ASCT was performed on 23 December, followed three days later by sequential autologous tandem CD19/20 CAR-T therapy. This optimized hinge tandem CAR targeting CD19 and CD20, incorporating both CD28 and 4-1BB co-stimulatory domains. Following CAR-T therapy, the patient developed immune effector cell-associated hematologic toxicity, which was successfully managed with G-CSF and supportive care. Notably, no cytokine release syndrome (CRS), immune effector cell-associated neurotoxicity syndrome (ICANS), severe infections, or other complications were observed.

**Figure 2:**
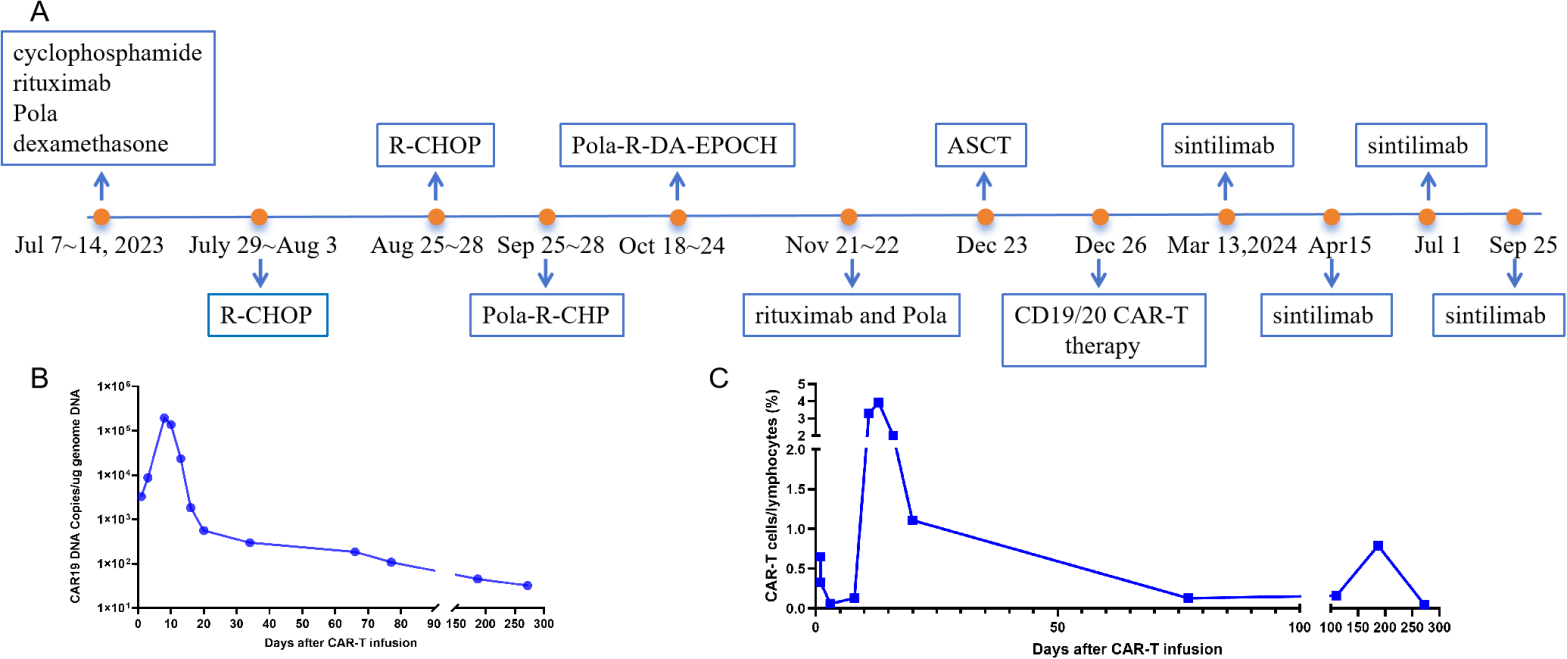
The timeline of the clinical treatment and evaluation of *in vivo* expansion of CAR-T cells. **(A)** the timeline of the clinical treatment. **(B)** CAR-T DNA copies were assessed by droplet digital polymerase chain reaction. **(C)** CAR-T cells were assessed by flow cytometry. R-CHOP (rituximab, cyclophosphamide, liposomal adriamycin, vincristine, dexamethasone); Pola-R-DA-EPOCH (polatuzumab, rituximab, etoposide, dexamethasone, cyclophosphamide, doxorubicin); Pola-R-CHP (polatuzumab, rituximab, cyclophosphamide, doxorubicin, dexamethasone. CAR-T, chimeric antigen receptor T-cell.

**Figure 3:**
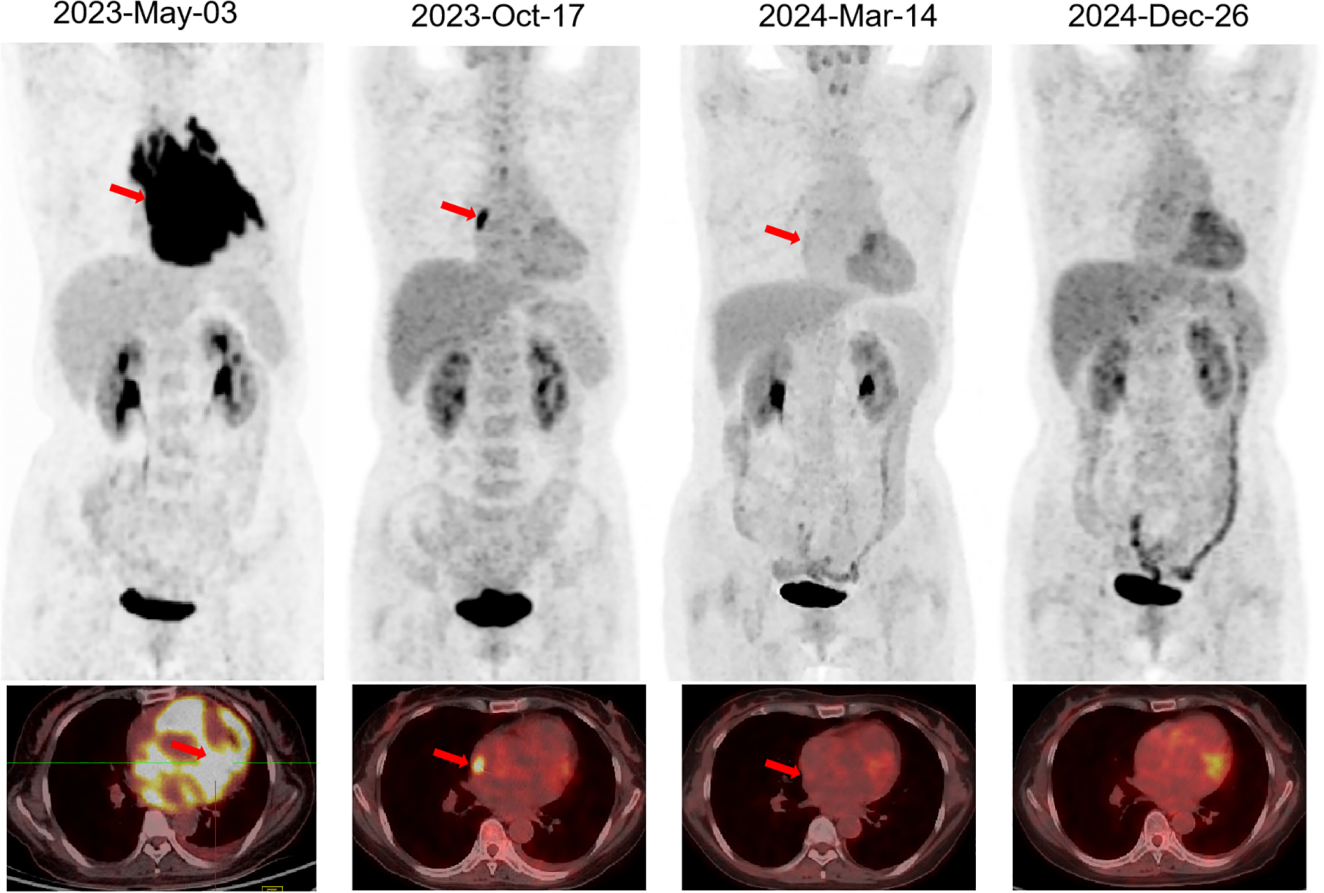
PET-CT evaluation of PCL. The arrow indicates the lesion. PET-CT, Positron Emission Tomography-Computed Tomography; PCL, primary cardiac lymphoma.

The PD-1 inhibitor sintilimab was administered approximately every two months as maintenance therapy for one year following CAR-T cell infusion (**Figure 2A**) (5). *In vivo* expansion of CAR-T cells was assessed by droplet digital polymerase chain reaction and flow cytometry, both of which demonstrated robust post-infusion expansion (**Figures 2B, C**). PET-CT evaluation in March 2024 confirmed that the patient had achieved CR with a Deauville score of 3. The patient showed resolution of symptoms. To date, the patient has survived for approximately two years since the onset of PCL and has maintained CR (Deauville score of 1 in December 2024) for 1 year since receiving the CAR-T infusion (**Figure 3**). She continues to undergo follow-up every 3 months. No severe infections or other adverse events occurred during maintenance therapy.

## Discussion

PCL is defined as lymphoma lesions involving only the heart and pericardium, large tumor masses identified at initial diagnosis, or main lesions located in the heart, with cardiac symptoms caused by myocardial infiltration of the lymphoma as the primary manifestation, which may be accompanied by metastatic features such as enlarged mediastinal lymph nodes and pleural effusion (4, 6). Compared to SCL, which occurs in approximately 25% of diffuse lymphomas, PCL accounts for less than 2% of all primary cardiac malignancies and approximately 0.5% of extranodal lymphomas (2). Historically, most PCL cases are of B-cell lineage, especially DLBCL. Follicular lymphoma, Burkitt lymphoma, and T-cell lineage PCL are sporadic. Currently, most reports on PCL are limited to individual case studies. PCL can occur at any age (ranging from 12 years to 86 years) and affects males more than females, with a male-to-female ratio of 2:1 (2). PCL can affect any part of the heart, with the most commonly involved sites being the right atrium, followed by the pericardium, right ventricle, left atrium, and left ventricle (2, 4). Epicardial and pericardial infiltration with pericardial effusion, as observed in our case, is typical. The clinical manifestations of PCL are nonspecific and largely depend on the location and progression of the lesion. Common clinical symptoms include dyspnea, congestive heart failure, precordial pain, arrhythmia, myocardial infarction, cardiac tamponade, and superior vena cava syndrome (7). Diagnosing PCL poses significant challenges due to its rarity and the nonspecific nature of its clinical presentation. The condition often mimics other cardiac pathologies, leading to potential delays in accurate diagnosis. Especially when pericardial effusion is the primary manifestation, it is essential to differentiate it not only from benign causes such as tuberculosis, viral infections, connective tissue diseases, hypothyroidism, and benign primary cardiac neoplasms, but also from other malignancies (2). In our case, symptoms such as chest tightness and back pain were initially suggestive of more common cardiovascular or musculoskeletal disorders. The diagnostic process was further complicated by the need to rule out infectious causes such as tuberculosis, which mimicked lymphoma and other tumors in clinical presentations and imaging findings of multiple serous cavity effusions.

ECG is limited in the detection of cardiac tumors. Echocardiography and CT scans can objectively reveal masses, pericardial effusion, pleural effusion, pericardial thickening or enlarged mediastinal lymph nodes; however, commonly non-specific findings (8). Advanced imaging techniques, including CMR and PET-CT, can clearly reveal the size, number, and location of lesions, which helps guide biopsy planning and determine the extent of surgery. However, the definitive diagnosis of PCL still relies primarily on histopathological and immunohistochemical analyses. Approximately half of PCL cases are diagnosed through open-chest surgical biopsy, while about one-third are confirmed via percutaneous biopsy. Samples are typically obtained from the tumor mass, lymph nodes, pericardium, and myocardium (1, 7, 9). Pericardiocentesis is a minimally invasive and clinically accessible method for obtaining cytological confirmation of lymphoma. However, its diagnostic yield is relatively low, often requiring multiple attempts. In contrast, direct biopsy or surgical intervention of intracardiac lesions carries a higher risk. In this case, centrifugation of pericardial fluid combined with the cell block (CB) technique may enhance the detection of lymphoma cells.

Due to its rarity and diagnostic challenges, the optimal treatment, maintenance, and consolidation strategies for PCL remain to be established. Current therapies are often adapted from treatment regimens for other lymphoma subtypes (6, 9). Treatment options for patients with PCL include chemotherapy, surgery, and ASCT, variably combined with radiotherapy. Chemotherapy is generally the first-line treatment, typically based on regimens for other types of lymphoma. CHOP is currently the most widely used chemotherapy regimen. Rituximab, a CD20 monoclonal antibody, is commonly added to the CHOP regimen to improve overall survival in CD20-positive patients with PCL. EPOCH and HO (doxorubicin, vincristine) have also been reported in the treatment of PCL. Approximately 80% of patients with PCL demonstrate a response to chemotherapy. Approximately 44.6% of patients with PCL and right heart obstruction undergo palliative tumor resection, which has not been shown to significantly improve survival (6, 9). However, when tumor obstruction leads to superior vena cava syndrome or cardiac rupture, emergency surgical intervention may offer a chance to save the patient’s life (10).

Due to its high aggressiveness, the prognosis of PCL is extremely poor (2). Early diagnosis of PCL is challenging, and many patients present with large tumor masses at initial detection. Common causes of death include cardiac rupture, massive pulmonary embolism, heart failure, and arrhythmia. An early epidemiological study enrolled 150 cases of PCL from 1973 to 2011 demonstrated that 1-, 3-, and 5-year survival rates were 59%, 41%, and 34%, respectively (11). A more recent study reviewed 121 cases of PCL reported in domestic and international literature from 2014 to 2024. The results showed that untreated patients had a median survival of less than one week. Among patients who received chemotherapy alone, 18.2% had died, with a median survival of 6 months, while 81.8% were alive with a median follow-up of 7 months. Among the 24 patients who underwent both surgery and chemotherapy, 7 died within one month, while 17 survived, with a median follow-up of 11 months. ASCT has only been reported in individual cases of PCL, with most demonstrating short-term efficacy (9). Only one report described ASCT, following palliative resection and chemotherapy, achieving sustained CR in a patient with DLBCL and right atrial involvement, who remained in CR at 1-year follow-up (12).

For PCL, early intervention is essential to alleviate symptoms and reduce the risk of fatal outcomes. Nevertheless, many patients exhibit poor tolerance to standard-dose chemotherapy, particularly regimens containing anthracyclines, which are known for their cardiotoxicity. In certain cases, dose reductions of chemotherapy agents such as cyclophosphamide and adriamycin are recommended to minimize cardiotoxic complications (13, 14). In this case, the patient was treated with a targeted combination chemotherapy regimen containing Pola, which is a novel antibody–drug conjugate (ADC) primarily used for the treatment of relapsed or refractory diffuse large B-cell lymphoma. Studies have shown that Pola demonstrates superior efficacy and tolerability compared to traditional chemotherapy (15). Pola functions by delivering cytotoxic agents directly to malignant B cells, thereby enhancing antitumor activity while minimizing off-target effects, including cardiotoxicity (15).

CAR-T therapy has brought transformative advancements in the treatment of B-cell lymphomas, overcoming limitations of ASCT, including its suboptimal efficacy and high relapse rate in relapsed or refractory DLBCL (16, 17). However, clinical trials often exclude lymphomas involving the heart; thus, the safety and efficacy of CAR-T therapy for cardiac lymphomas remain unknown. To our knowledge, fewer than 10 reported cases of CAR-T therapy for cardiac lymphoma have been reported, most of which involved SCL, with the first reported in 2020 (18–21). The CARTCO (NCT05130489) study conducted at University College London Hospital described the feasibility of CAR-T therapy in four cases of cardiac lymphoma; however, it did not distinguish between PCL and SCL. Three cases achieved PR at months 3–6, and 1 achieved CR at month 3 of follow-up with CAR-T therapy (21). While cardiotoxicity was reported in all four cases, no fatalities were attributed to it. Common cardiac toxicities following CAR-T therapy include hypotension, left ventricular systolic dysfunction, arrhythmias, and biomarker abnormalities (22, 23). The severity of CRS may be associated with the occurrence or exacerbation of cardiac complications (24). The underlying pathophysiologic mechanism remains unclear and is thought to involve systemic inflammatory response-mediated effects on the myocardium and cardiac conduction system (19, 22, 23, 25). Although CAR-T therapy has cardiotoxic adverse effects, emerging reports in recent years have described its use for lymphomas with coexisting cardiomyopathy, including cases beyond SCL, suggesting the feasibility of CAR-T therapy for treating lymphomas involving the heart (18–21).

Building on our center’s experience with ASCT and sequential CAR-T therapy for refractory or relapsed aggressive B-cell non-Hodgkin lymphoma, we report for the first time the successful application of tandem CD19/20 CAR-T therapy combined with ASCT in the treatment of PCL. Our previous studies showed that this treatment strategy demonstrated higher response rates, progression-free survival, and overall survival compared to CAR-T therapy alone, particularly in high-risk subtypes of B-NHL (26, 27).

The combination treatment led the patient to achieve CR at month 3 and maintain sustained CR for over one year, with an overall survival of approximately two years since the onset of PCL. Although current studies suggest that most patients with PCL, including the present case, are sensitive to chemotherapy, it is associated with significant cumulative toxicity, and sustained long-term remission is rarely reported (9, 28). Surgical intervention has not been shown to prolong survival and may contribute to poor outcomes, including increased risks of metastasis and mortality (29, 30). Reports on patients receiving ASCT are limited, with most failing to demonstrate durable efficacy (31). Likewise, individual cases of CAR-T therapy for cardiac lymphoma have not shown evidence of long-term remission (20, 21). These findings highlight the potential of combining ASCT with tandem CD19/20 CAR-T therapy as a promising treatment strategy for achieving durable remission in patients with high-risk PCL, warranting further investigation in larger studies.

The synergistic effect of ASCT and sequential CAR-T therapy may be attributed to immune reconstitution induced by ASCT, which potentially promotes a favorable cytokine milieu that enhances T-cell trafficking, facilitates the recruitment of innate immune cells, and suppresses immunosuppressive components within the tumor microenvironment (27, 32, 33). We found that using ADC drugs such as Pola for bridging therapy not only effectively improved PCL but also showed no adverse effects on subsequent CAR-T cell manufacturing, functionality, or persistence. To optimize the comprehensive management of CAR-T therapy, we employed a PD-1 inhibitor as maintenance therapy following CAR-T infusion. This approach supported sustained CAR-T proliferation. No severe adverse events were reported. This strategy underscores the importance of optimizing the entire CAR-T therapy process—including bridging and maintenance—by balancing anti-tumor efficacy, CAR-T cell functionality and persistence, tolerability, and safety, thereby improving patient outcomes. Furthermore, the management algorithm of CD19-targeted CAR-T therapy for B-NHL with cardiac involvement emphasizes the importance of close monitoring in an intensive care unit and decision-making by an expert MDT (21).

This study has several limitations. First, this is a single-case report, and the findings may not be generalizable to the broader population of patients with PCL. Second, due to the rarity and complexity of PCL, therapeutic decisions were based on clinical judgment and experience rather than established guidelines. Third, long-term outcomes beyond the second year remain unknown and require ongoing follow-up.

## Conclusion

Taken together, PCL is an exceedingly rare malignancy with a poor prognosis. Timely diagnosis is critical to enable early initiation of treatment. Robust evidence supporting an optimal treatment strategy for PCL remains limited due to its rarity and diagnostic challenges. This case highlights both the diagnostic challenges of PCL and the potential efficacy and safety of innovative treatments—such as ADC-based chemotherapy, ASCT and sequential CAR-T therapy—in managing this rare and aggressive malignancy. Additionally, this is the first reported case utilizing a combination of ASCT and sequential CAR-T therapy, along with a detailed algorithm for optimizing the entire CAR-T therapy process. This combination therapy resulted in enhanced response, reduced toxicity, and sustained remission. Further validation through long-term follow-up and expanded case studies, as well as mechanistic studies investigating interactions between the cardiac lymphoma microenvironment and cellular therapies, is warranted.

## Data Availability

The raw data supporting the conclusions of this article will be made available by the authors, without undue reservation.
