# Supplementary material for: Durable response of primary cardiac lymphoma after autologous stem cell transplantation and sequential CAR-T therapy: a case report and literature review
Source: Front Immunol. 2025 Aug 28;16:1581654. doi: 10.3389/fimmu.2025.1581654 (PMC12423075; doi:10.3389/fimmu.2025.1581654)
Supplement: Supplementary file 1 [file Table1.docx]

Table S1 detailed manufacture information of the drugs, antibodies, etc used in the case report

| drugs, antibodies, etc | manufacture information |
| --- | --- |
| CD19/20 CAR-T | Hebei Senlang Biotechnology Inc., Ltd. |
| Cyclophosphamide | Baxter Oncology GmbH |
| Dexamethasone | Ma'anshan Fengyuan Pharmaceutical Co., Ltd. |
| Doxorubicin | EBEWE Pharma GmbH Nfg. KG |
| Etoposide | Jiangsu Hengrui Pharmaceuticals Co., Ltd |
| Granulocyte colony-stimulating factor | Hangzhou Jiuyuan Gene Engineering Co., Ltd |
| Liposomal adriamycin | Changzhou Jinyuan Pharmaceutical Manufacturing Co., Ltd |
| Micafungin | Jiangsu Hansoh Pharmaceutical Group Co., Ltd |
| Polatuzumab vedotin | F. Hoffmann-La Roche Ltd. |
| Rituximab | F. Hoffmann-La Roche Ltd. |
| Sintilimab | BeOne Medicines Ltd. |
| Trimethoprim-sulfamethoxazole | PKU Healthcare Corp., Ltd |
| Vincristine | Hangzhou Minsheng Pharmaceutical Co., Ltd. |

Table S2: full CBC blood assessments and liver functionality of the patient for before and after sequential CAR-T following ASCT

| assessments | before sequential CAR-T following ASCT | 7 days after sequential CAR-T following ASCT | 30 days after sequential CAR-T following ASCT |
| --- | --- | --- | --- |
| White Blood Cell | 2.30*10^9/L | 0.04*10^9/L | 3.74*10^9/L |
| Neutrophil | 1.29*10^9/L | 0*10^9/L | 0.75*10^9/L |
| Hemoglobin | 79.0g/L | 79g/L | 92g/L |
| Platelet | 172*10^9/L | 31*10^9/L | 89*10^9/L |
| Alanine Aminotransferase | 7U/L | 18U/L | 10U/L |
| Aspartate Aminotransferase | 15U/L | 23U/L | 24U/L |
| Total Bilirubin | 3.3umol/L | 5.3umol/L | 6.2umol/L |
| Indirect Bilirubin | 1.7umol/L | 3.0umol/L | 4.2umol/L |
| Alkaline Phosphatase | 76U/L | 104U/L | 137U/L |
| γ Glutamyl Transferase | 32U/L | 201U/L | 175U/L |
| Lactate Dehydrogenase | 288U/L | 262U/L | 229U/L |
