# Supplementary figures and images for: Durable response of primary cardiac lymphoma after autologous stem cell transplantation and sequential CAR-T therapy: a case report and literature review
Source: Front Immunol. 2025 Aug 28;16:1581654. doi: 10.3389/fimmu.2025.1581654 (PMC12423075; doi:10.3389/fimmu.2025.1581654)

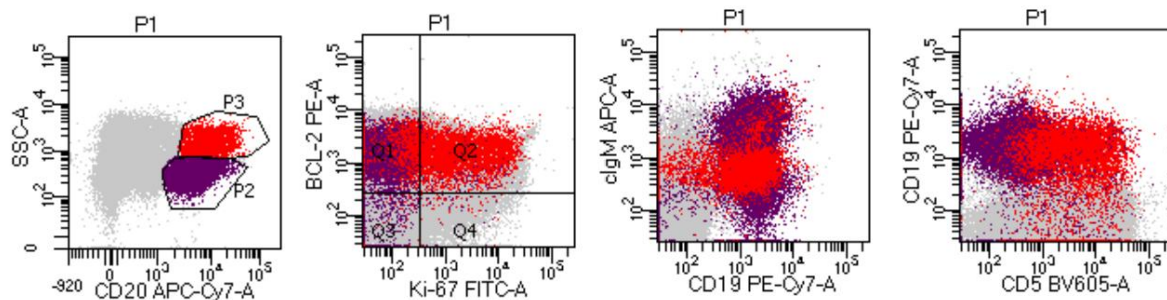

Tube: 5/45/Ki-67/BCL-2/19/clgM/20

| Population | #Events   | %Parent | %Total |
|------------|-----------|---------|--------|
| All Events | 1,000,000 | ####    | 100.0  |
| Time       | 1,000,000 | 100.0   | 100.0  |
| Total      | 903,026   | 90.3    | 90.3   |
| P1         | 892,705   | 98.9    | 89.3   |
| P2         | 26,985    | 3.0     | 2.7    |
| P3         | 5,090     | 0.6     | 0.5    |
| Q1         | 985       | 19.4    | 0.1    |
| Q2         | 3,998     | 78.5    | 0.4    |
| Q3         | 39        | 0.8     | 0.0    |
| Q4         | 68        | 1.3     | 0.0    |

Supplement: Supplementary file 2 [file DataSheet1.pdf]
